# Supplementary material for: The TNFR Wengen regulates the FGF pathway by an unconventional mechanism
Source: Nat Commun. 2023 Sep 21;14:5874. doi: 10.1038/s41467-023-41549-3 (PMC10514202; doi:10.1038/s41467-023-41549-3)
Supplement: Supplementary file 1 — Supplementary Information [file 41467_2023_41549_MOESM1_ESM.pdf]

Supplementary Information for

The TNFR Wengen regulates the FGF pathway by an unconventional mechanism

Annalisa Letizia[1], Maria Lluisa Espinàs[1], Panagiotis Giannios[1,2], Marta Llimargas[1, *]

[1] Department of Cells and Tissues
Institut de Biologia Molecular de Barcelona, IBMB-CSIC.
Parc Científic de Barcelona
Baldiri Reixac, 10-12. 08028 Barcelona, Spain
[2] Institute for Research in Biomedicine (IRB Barcelona),
The Barcelona Institute of Science and Technology (BIST),
Baldiri Reixac 10, 08028, Barcelona, Spain

* Correspondence: mlcbmc@ibmb.csic.es

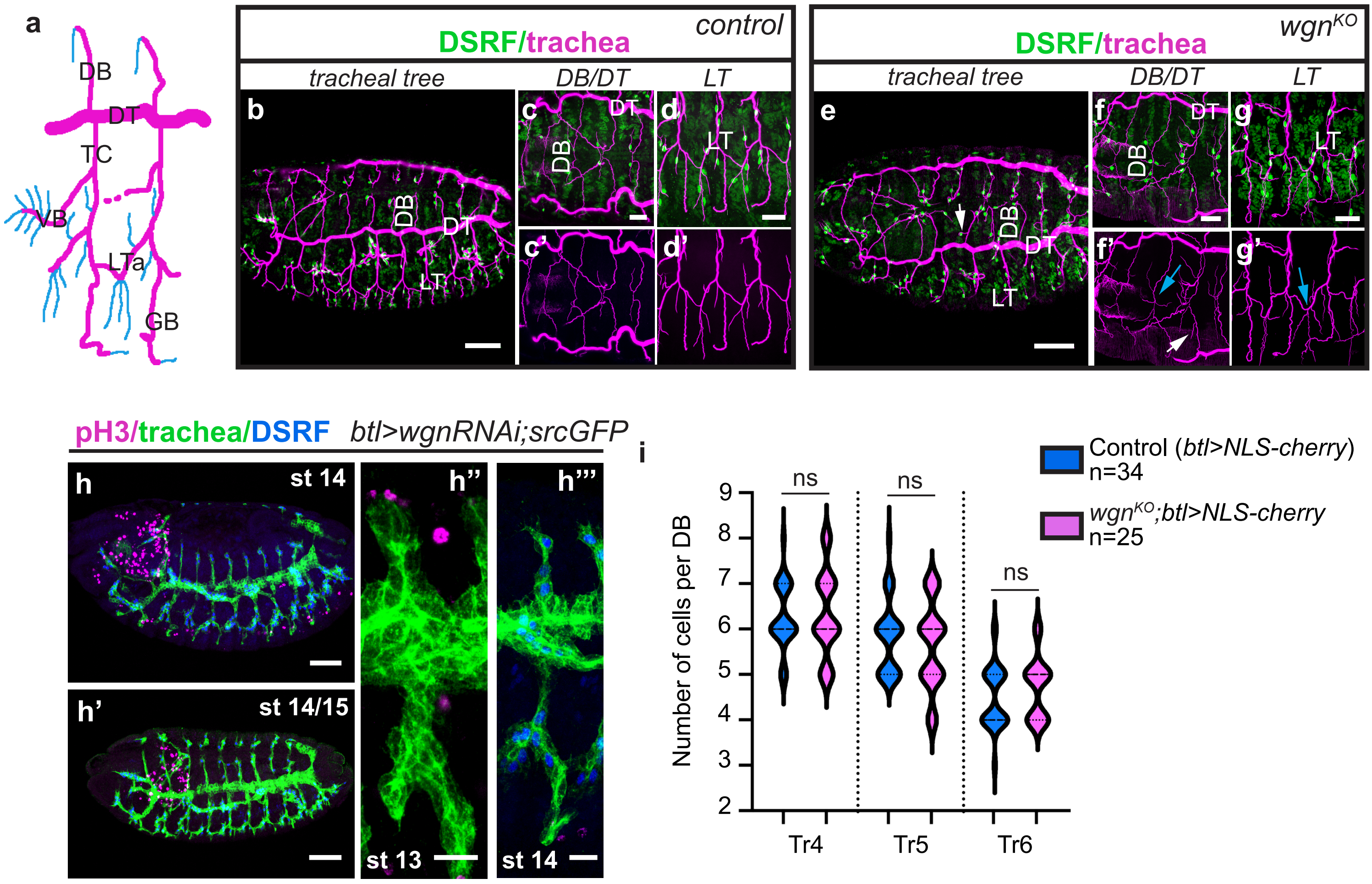

Supplementary Figure 1: Analysis of the tracheal requirements of TNFR-wgn

a Schematic diagram of 2 tracheal metameres in a lateral view showing the main tracheal branches in magenta: dorsal branch (DB), dorsal trunk (DT), transverse connective (TC), lateral trunk anterior (LTa), ganglionic branch (GB), visceral branch (VB). Fine blue lines indicate the terminal branches.

b-g Control or TNFR-wgn mutant embryos at late stage 15-stage 16 stained with CBP to visualise the tracheal tubes (magenta) and with DSRF to visualise the terminal cells (green). Different views show an excess of terminal cells in TNFR-wgn mutants compared to control, which generate excess of terminal branches (white arrows point to terminal branches in the DB stalk). In spite of these defects, the general branching pattern and the fusion events (LT, DB, blue arrows in f', g') are not affected.

h Embryos expressing TNFR-wgn RNAi in the tracheal system at the indicated stages stained with pH3 to detect cell division (magenta), DSRF to identify the terminal cells (blue) and GFP to visualise the tracheal system (green). Note the absence of pH3 staining in the trachea and in terminal cells at stage 13 (just before the differentiation of terminal cells) and at stage 14 (once terminal cells are specified).

i Quantification of the number of cells per DB in the metameres indicated (Tr 4,5 and 6) in control and TNFR-wgn mutants. Cell numbers were counted using the nuclear pattern of NLS-cherry expressed in the trachea. Violin plots indicate the distribution of the number of cells. n indicates the number of embryos analysed. ns not significant, non-parametric Mann-Whitney two-tailed test. No significant differences were observed in tracheal metamere 4 (p=0.89), tracheal metamere 5 (p=0.55) and tracheal metamere 6 (p=0.25)

Scale bar: b,e, h, h' 50 μm; c,d,f,g 20 μm; h'', h''' 10 μm.

Source data and details of statistical tests used and p values are provided as a Source Data file.

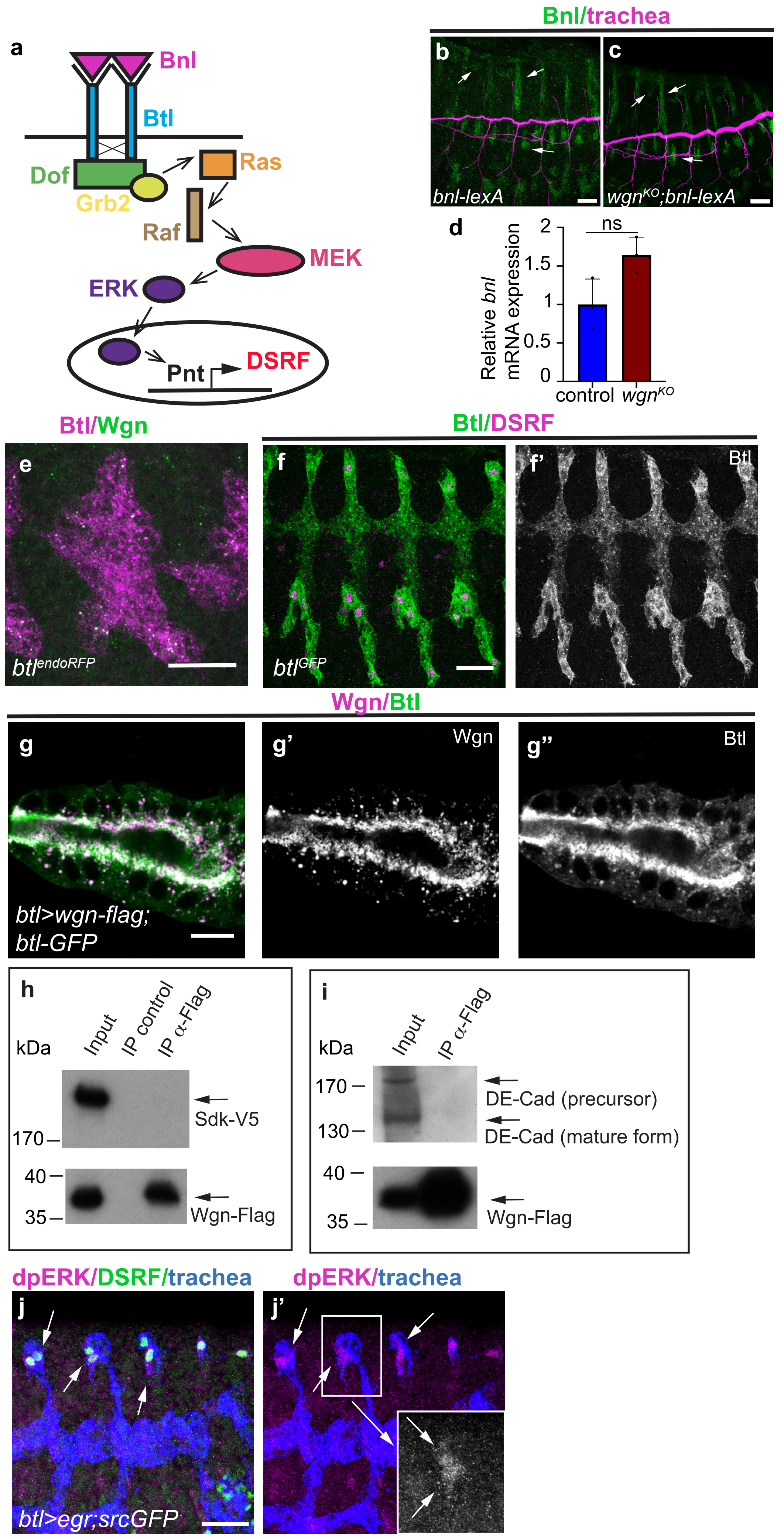
a
Bnl
Btl
Dof
Grb2
Ras
Raf
MEK
ERK
Pnt
DSRF
Bnl/trachea
b
c
bnl-lexA
wgnKO;bnl-lexA
d
ns
Relative bnl mRNA expression
2
1.5
1
0.5
0
control
wgnKO
Btl/Wgn
e
btlendoRFP
Btl/DSRF
f
f'
Btl
btlGFP
Wgn/Btl
g
g'
Wgn
g''
Btl
btl>wgn-flag;
btl-GFP
h
kDa
Input
IP control
IP α-Flag
Sdk-V5
170
40
35
Wgn-Flag
i
kDa
Input
IP α-Flag
170
130
DE-Cad (precursor)
DE-Cad (mature form)
40
35
Wgn-Flag
dpERK/DSRF/trachea
j
btl>egr;srcGFP
dpERK/trachea
j'

Supplementary Figure 2: FGFR-Btl and TNFR-Wgn

a Scheme of the FGFR-Btl pathway

b,c Lateral views of stage 14 embryos stained with RFP (green) to visualise *bnl-lexA/lexO-CAAXmcherry* reporter expression and with CBP to visualise the tracheal tree. Note Bnl expression in control and *TNFR-wgn* mutants in cells close to trachea (white arrows).

d *FGF-bnl* transcriptional levels were normalized to RpL23. Levels of *FGF-bnl in TNFR-wgn* mutants were normalised to control. n=3, Bars show SD of mean. ns not significant (p=0.57), two-tailed unpaired t test with Welch's correction.

e,f Lateral views of stage 11/12 (e) and 14 (f) embryos showing the pattern of FGFR-Btl accumulation using tagged alleles. Note the increased accumulation of FGFR-Btl at the tips, in DSRF expressing cells, at stage 14 (f').

g Salivary gland expressing *TNFR-wgn-Flag* and *FGFR-btl-GFP* showing the accumulation of the two proteins in common intracellular vesicles

h,i Control co-immunoprecipitation experiments. Western blot using either αV5 or DCAD2 antibody (upper left and right panels respectively) and αFlag (lower panels) of extracts of third-instar larvae salivary glands expressing either *TNFR-wgn-flag* and *Sdk-V5* (left panels) or *TNFR-wgn-flag* and *shotgun* (*shg, DE-Cad)* (right panels). Extracts were immunoprecipitated using αFlag antibodies or a control antibody (αAbd-B). Input corresponds to 10 % (*Sdk-V5*) and 5% (*DE-Cad*) of the immunoprecipitated material.

j Lateral views of a stage 14 embryo stained with dpERK (magenta), DSRF to identify the terminal cells (green) and GFP to visualise the tracheal system (blue). Note that dpERK accumulates in the extra terminal cells (white arrows, inset shows the dpERK chanel).

Scale bar: b,c,e,f,j 20 μm; g 10 μm.

Source data and details of statistical test used and p value are provided as a Source Data file.

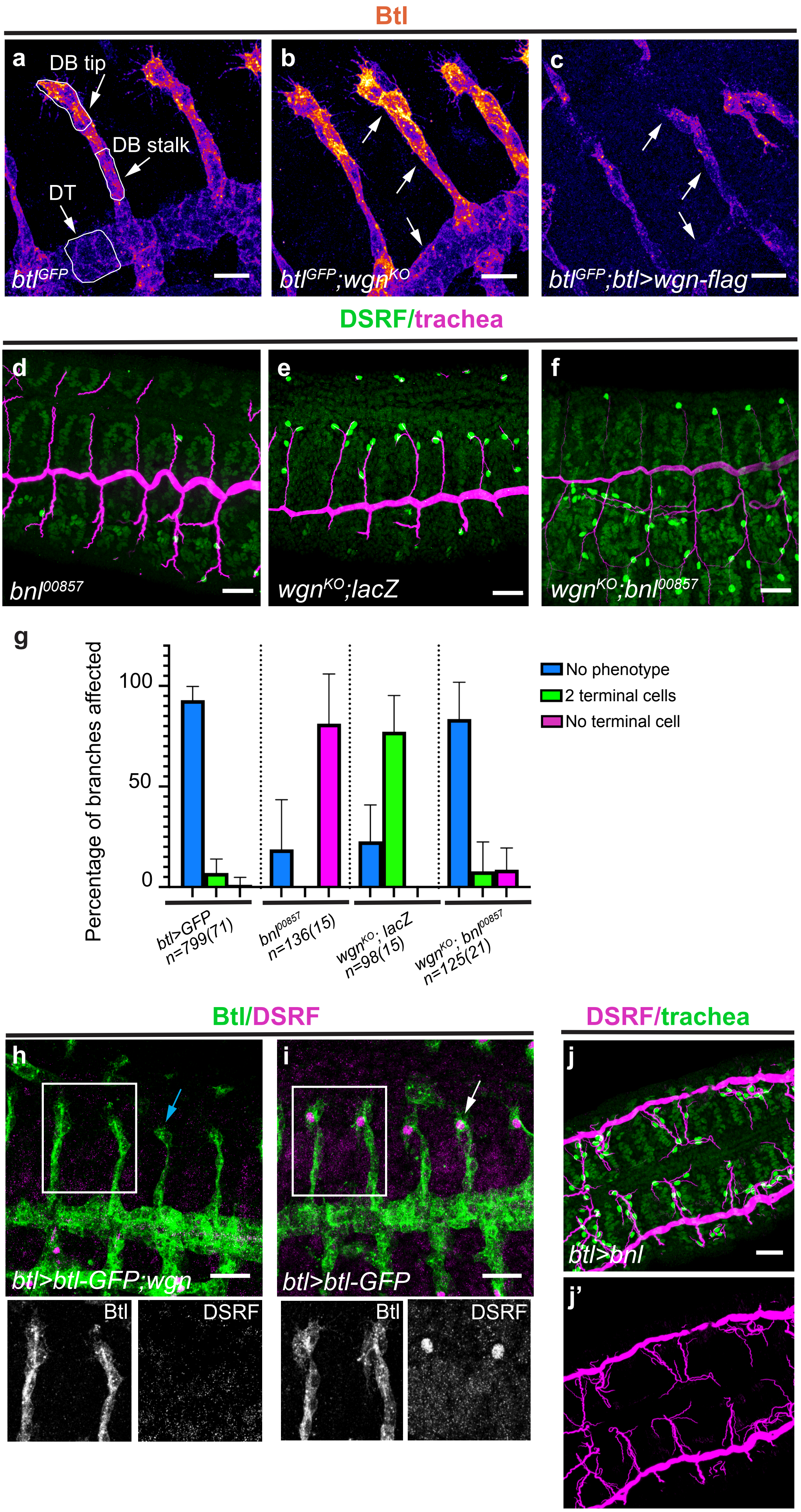

Btl
a
DB tip
DB stalk
DT
btl^GFP
b
btl^GFP;wgn^KO
c
btl^GFP;btl>wgn-flag
DSRF/trachea
d
bnl^00857
e
wgn^KO;lacZ
f
wgn^KO;bnl^00857
g
Percentage of branches affected
100
50
0
btl>GFP n=799(71)
bnl^00857 n=136(15)
wgn^KO; lacZ n=98(15)
wgn^KO; bnl^00857 n=125(21)
No phenotype
2 terminal cells
No terminal cell
Btl/DSRF
h
btl>btl-GFP;wgn
Btl
DSRF
i
btl>btl-GFP
Btl
DSRF
DSRF/trachea
j
btl>bnl
j'

Supplementary Figure 3: Regulation of FGFR-Btl levels and activity of FGFR-Btl pathway in terminal cell specification

a-c Lateral views of stage 14 embryos showing a representative example of the accumulation of FGFR-Btl in the different genotypes indicated. Regions used for quantifications in Figure 6a are shown. The fluorescence intensity of *FGFR-Btl* is shown in heat maps. (DB, dorsal branch; DT, dorsal trunk)
d-f Dorso-lateral views of stage 15 embryos of the indicated genotypes stained with DSRF (green) and CBP as a tracheal marker (magenta).
g Quantification of the percentage of dorsal branches that show the indicated phenotypes in the different genotypes. Note the effects of decreasing *FGF-bnl* levels in terminal cell differentiation. Note the rescue of extra terminal cells in *TNFR-wgn* mutants when the *FGF-bnl* dose is reduced. Bars show SD of mean. n, number of DBs analysed, in brackets number of embryos analysed.
h,i Lateral views of stage 14/15 embryos of the indicated genotypes stained with GFP (green) to visualise the *FGFR-btl-GFP* overexpression and with DSRF to detect the terminal cells. When *TNFR-wgn* is overexpressed no terminal cells are specified (blue arrow in h) in spite of presence of overexpressed *FGFR-btl*. *FGFR-btl* overexpression does not lead to extraterminal cells (white arrow in i).
j Lateral view of a stage 14/15 embryo expressing FGF-Bnl in tracheal cells stained with DSRF (magenta) and CBP (green). FGF-Bnl tracheal expression leads to many extra terminal cells.
Scale bar: a-c 10 μm; d-f,h-j 20 μm.

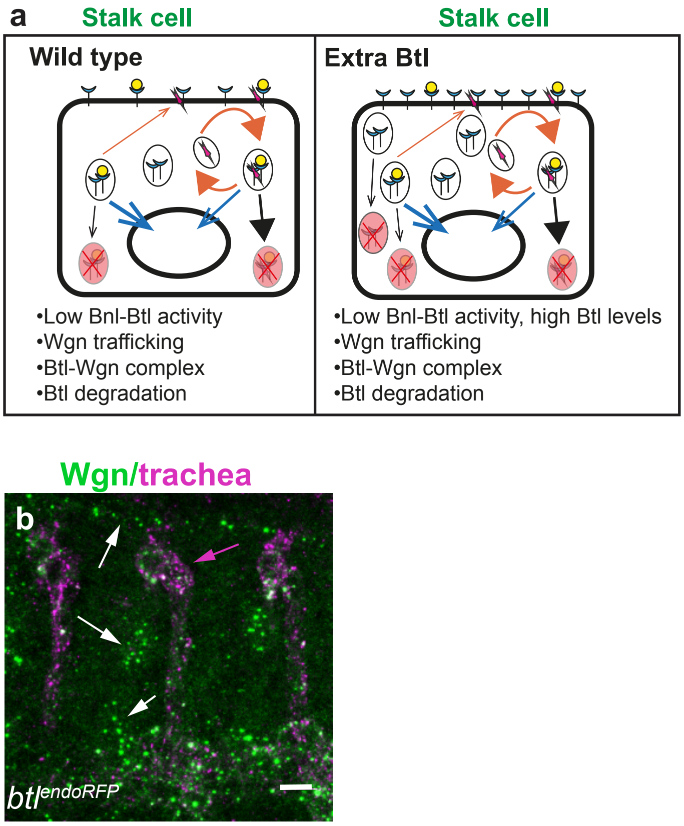

Supplementary Figure 4: TNFR-Wgn in other tissues

a Model. The stalk cell receives low levels of FGF-Bnl ligand, leading to a weak activation of the pathway. This, combined with the negative effect of TNFR-Wgn, prevents DSRF activation. When FGF-Btl is overexpressed, in spite of increased levels of FGFR-Btl, TNFR-Wgn exerts its negative effect and the activating FGF-Bnl ligand remains limitant, preventing ectopic DSRF activation.

b Lateral view of a stage 14 embryo stained with αWgn (green) and with RFP to visualise the tracheal cells (magenta, magenta arrow). Note the presence of TNFR-Wgn in intracellular vesicles in tissues other than the trachea (white arrows).

Scale bar: b 20 μm

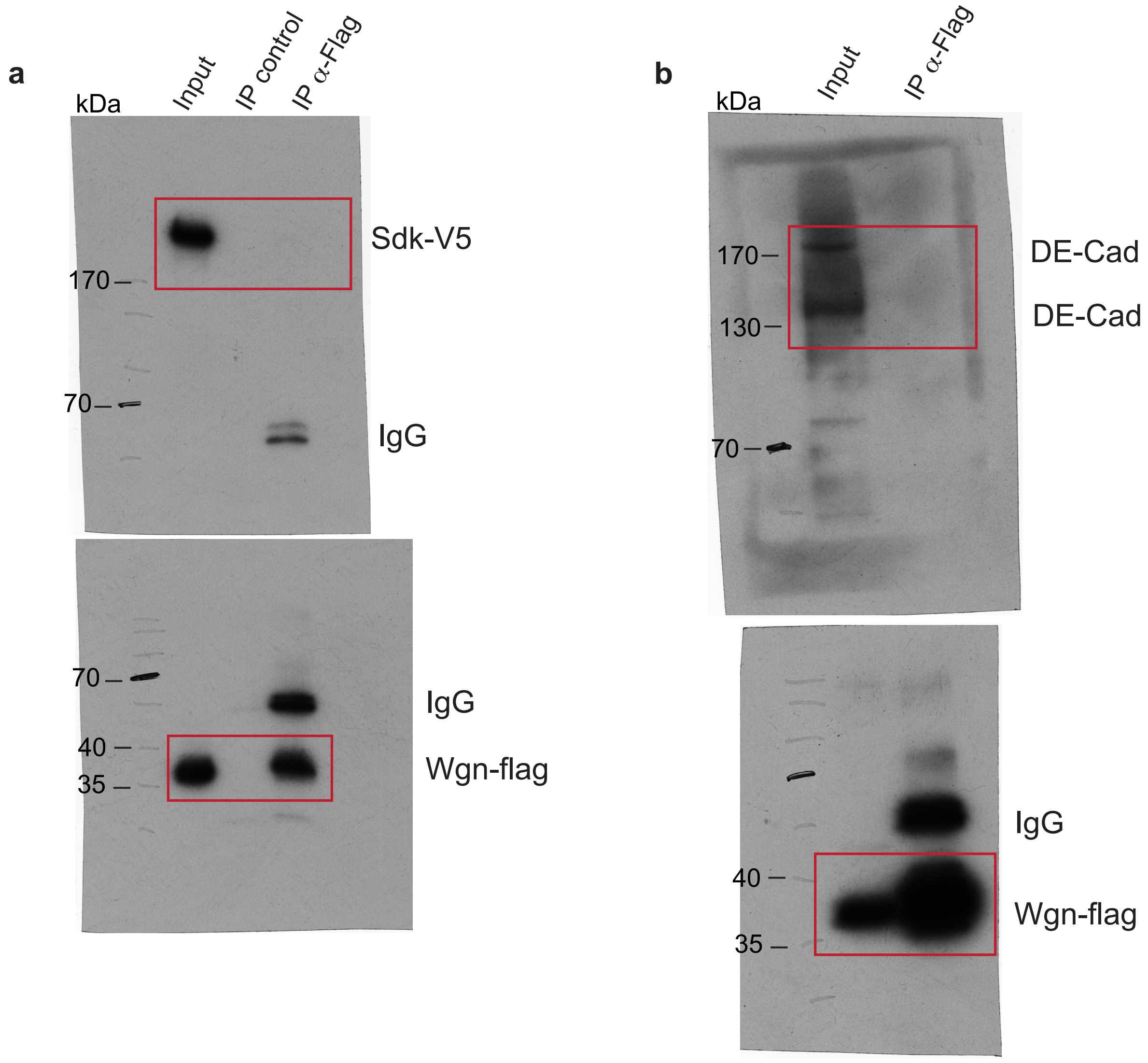

Supplementary Figure 5: Uncropped and unprocessed scans of blots

a Uncropped and unprocessed scans of Western blots using αV5 antibody (upper panel) and αFlag (lower panel) corresponding to Supplementary Figure 2h.

b Uncropped and unprocessed scans of Western blots using DCAD2 antibody (upper panel) and αFlag (lower panel) corresponding to Supplementary Figure 2i.

Red boxes correspond to the image shown in Supplementary Figure 2h,i.
