# Supplementary material for: The TNFR Wengen regulates the FGF pathway by an unconventional mechanism
Source: Nat Commun. 2023 Sep 21;14:5874. doi: 10.1038/s41467-023-41549-3 (PMC10514202; doi:10.1038/s41467-023-41549-3)
Supplement: Supplementary file 3 — Reporting Summary [file 41467_2023_41549_MOESM3_ESM.pdf]

nature portfolio

Corresponding author(s): Marta Llimargas

Last updated by author(s): 2023/08/31

Reporting Summary

Nature Portfolio wishes to improve the reproducibility of the work that we publish. This form provides structure for consistency and transparency in reporting. For further information on Nature Portfolio policies, see our Editorial Policies and the Editorial Policy Checklist.

Statistics

For all statistical analyses, confirm that the following items are present in the figure legend, table legend, main text, or Methods section.

n/a	Confirmed	
☐	☒	The exact sample size (n) for each experimental group/condition, given as a discrete number and unit of measurement
☐	☒	A statement on whether measurements were taken from distinct samples or whether the same sample was measured repeatedly
☐	☒	The statistical test(s) used AND whether they are one- or two-sided<br>*Only common tests should be described solely by name; describe more complex techniques in the Methods section.*
☒	☐	A description of all covariates tested
☐	☒	A description of any assumptions or corrections, such as tests of normality and adjustment for multiple comparisons
☐	☒	A full description of the statistical parameters including central tendency (e.g. means) or other basic estimates (e.g. regression coefficient) AND variation (e.g. standard deviation) or associated estimates of uncertainty (e.g. confidence intervals)
☐	☒	For null hypothesis testing, the test statistic (e.g. F, t, r) with confidence intervals, effect sizes, degrees of freedom and P value noted<br>*Give P values as exact values whenever suitable.*
☒	☐	For Bayesian analysis, information on the choice of priors and Markov chain Monte Carlo settings
☒	☐	For hierarchical and complex designs, identification of the appropriate level for tests and full reporting of outcomes
☒	☐	Estimates of effect sizes (e.g. Cohen's d, Pearson's r), indicating how they were calculated

Our web collection on statistics for biologists contains articles on many of the points above.

Software and code

Policy information about availability of computer code

Data collection	Leica AF (version 2011.4.1) software was used for image acquisition
Data analysis	The images were imported and processed using Fiji (ImageJ2 version 2.9.0/1.53t) for measurements and quantifications. Images were assembled into figures using Photoshop 2020 and Illustrator 2020 (Adobe Inc).<br>Statistical analyses were performed using Graph Pad Prism 9.5.0 (Graph Pad Software Inc., La Jolla, CA, USA). Differences were considered significant when p < 0.05

For manuscripts utilizing custom algorithms or software that are central to the research but not yet described in published literature, software must be made available to editors and reviewers. We strongly encourage code deposition in a community repository (e.g. GitHub). See the Nature Portfolio guidelines for submitting code & software for further information.

Data

Policy information about availability of data

All manuscripts must include a data availability statement. This statement should provide the following information, where applicable:

- Accession codes, unique identifiers, or web links for publicly available datasets
- A description of any restrictions on data availability
- For clinical datasets or third party data, please ensure that the statement adheres to our policy

Flybase database was used (http://flybase.org/). The authors declare that all data supporting the findings of this study are available within the article and its supplementary information files and in the Source Data File.

Research involving human participants, their data, or biological material

Policy information about studies with human participants or human data. See also policy information about sex, gender (identity/presentation), and sexual orientation and race, ethnicity and racism.

Reporting on sex and gender	Not applicable
Reporting on race, ethnicity, or other socially relevant groupings	Not applicable
Population characteristics	Not applicable
Recruitment	Not applicable
Ethics oversight	Not applicable

Note that full information on the approval of the study protocol must also be provided in the manuscript.

Field-specific reporting

Please select the one below that is the best fit for your research. If you are not sure, read the appropriate sections before making your selection.

☒ Life sciences ☐ Behavioural & social sciences ☐ Ecological, evolutionary & environmental sciences

For a reference copy of the document with all sections, see nature.com/documents/nr-reporting-summary-flat.pdf

Life sciences study design

All studies must disclose on these points even when the disclosure is negative.

Sample size	Sample sizes for quantifications are indicated in the Figures and corresponding legends, and represent a sufficient size to obtain significative statistical values. Sample sizes corresponding to image analyses were chosen by visual inspection of the data, and correspond to more than 3 acquisitions/observations
Data exclusions	No data exclusions were applied, except in cases in which the data had insufficient quality or was damaged.
Replication	Experiments were typically repeated at least 3 times and were always reproducible
Randomization	Individual flies/embryos/larvae were chosen randomly amongst those allocated into experimental groups for each particular parameter. Experimental groups were defined by genotype using Drosophila balancer chromosomes or phenotypic markers
Blinding	Group allocation was determined by genotype and reseachers were not blind to group allocation, as the genotypes need to be visible for proper allocation. Blinding was performed for quantifications. We obtained multiple images for each experiment (and from independent experiments) and they were analysed by at least two authors.

Reporting for specific materials, systems and methods

We require information from authors about some types of materials, experimental systems and methods used in many studies. Here, indicate whether each material, system or method listed is relevant to your study. If you are not sure if a list item applies to your research, read the appropriate section before selecting a response.

Materials & experimental systems

n/a	Involved in the study
☐	☒ Antibodies
☒	☐ Eukaryotic cell lines
☒	☐ Palaeontology and archaeology
☐	☒ Animals and other organisms
☒	☐ Clinical data
☒	☐ Dual use research of concern
☒	☐ Plants

Methods

n/a	Involved in the study
☒	☐ ChIP-seq
☒	☐ Flow cytometry
☒	☐ MRI-based neuroimaging

Antibodies

Antibodies used

The antibodies used in this study are listed in the methods section. The following primary antibodies were used: rabbit anti-Arl8 (1:100, AB_2618258, DSHB); mouse anti-Wgn (1:200, kindly provided by K. Basler); rabbit anti-DSRF (1:400, kindly provided by N. Martín, Prof. J.Casanova lab); goat anti-GFP (1:600, ab6673, AbCam); rabbit anti-GFP (1:600, A11122, ThermoFisher Scientific–Invitrogen); mouse anti-flag (for IF 1:200, for WB 1:10000, A00187, clone 5A8E5, GenScript); mouse anti-dpERK (1:100, M8159, clone MAPK-YT, Sigma); rabbit anti-btl (1:2000 for WB, kindly provided by J. Casanova lab); rabbit anti-Dys (1:500, kindly provided by L. Jiang); rabbit anti-Rab7 (1:1000, kindly provided by T. Tanaka. 58); chicken anti-b Gal (1:600, ab9361, AbCam); rabbit anti-RFP (1:300, ab62341, AbCam); mouse anti-Abd-B (1A2E9, AB_528061, DSHB); rabbit anti-Cleaved Drosophila Dcp1 (1:100, 9578S, Cell Signaling Technology); rabbit anti-egr (1:50, described in 59); rabbit anti-trh (1:100, kindly provided by J. Casanova); rabbit anti-P-Histone H3 (1:100, 9701S, Cell Signaling Technology); rat anti-Dcad2 (for WB 1:4000, AB_528120, DSHB); mouse anti-V5 (for WB 1:8000, R960-25, clone SV5-Pk1, ThermoFisher Scientific – Invitrogen); Chitin Binding Probe fluorescently labelled CBP (1:300, kindly provided by N. Martín, Prof. J.Casanova lab). The following secondary antibodies were used at 1:300: Cy3 AffiniPure Donkey Anti-Mouse IgG (H+L), 715-165-150; Cy3 AffiniPure Donkey Anti-Chicken IgY (IgG) (H+L), 703-165-155; Cy2 AffiniPure Donkey Anti-Chicken IgY (IgG) (H+L), 703-225-155; Cy5 AffiniPure Donkey Anti Rabbit IgG (H+L), 711-175-152; Cy5 AffiniPure Donkey Anti-Goat IgG (H+L), 705-175-147; Cy3 AffiniPure Goat Anti-Mouse IgG (H+L), 115-165-003; Cy2 AffiniPure Goat Anti Rabbit IgG (H+L), 111-225-144; Cy5 AffiniPure Goat Anti-Mouse IgG (H+L), 115-175-146; Cy5 AffiniPure Goat Anti-Rabbit IgG (H+L), 111-175-144 (Jackson Inmunoresearch) and Alexa Fluor® 647 Donkey anti mouse, A31571; Alexa Fluor Plus 488 Donkey anti-Goat IgG (H+L), A32814 (Life Technologies /Thermofisher Scientific).

Validation

All antibodies in this work have been validated by the publications described in Developmental Studies Hybridoma Bank, Abcam, Thermofisher, GenScript, Sigma, Cell Signaling Technology, Jackson immunoresearch or in the references indicated in the manuscript, and are of regular use in the Drosophila community

Animals and other research organisms

Policy information about studies involving animals; ARRIVE guidelines recommended for reporting animal research, and Sex and Gender in Research

Laboratory animals

We used Drosophila melanogaster embryos and larvae of different genotypes specified in the manuscript. Adult flies were use 1-15 days after hatching. The fly strains used are the following: stocks obtained from the Bloomington Drosophila Stock Center: y1w1118 (# 6598), UASCherry.
NLS (#38425), UASwgn-Trip.HMC03962 (#55275), UAS-Traf2 (#58991), UAS-Tak1 DN (#58811), UAS-hepCA (#9306), UASbskDN (#6409), tre-GFP (#59010), UAS-FyveGFP-myc (#42716), Rab4EYFP (#62542), UAS-RasV12 (#4847), UAS-btIGFP (#41802), UASbtl. (#29046), UAS-btl RNAi (#60013), bn100857 (#6384), UAS-bnl (#64232), UAS-shrubGFP (#32559); stocks from the Vienna Drosophila Resource Center: UASwgn-VSH330339 (#330339), UAS-Traf2 RNAi (#16125), UAS-hepRNAi (#109277), btIGFP (#318302). btl-Gal4 was kindly provided by Prof. S. Hayashi; fkh-Gal4 was kindly provided by Prof. D. Andrew; UAS-bnIGFP, bnl-LexA, lexOCAAXmcherry,
bnlendoGFP, btlendoRFP, were kindly provided by Prof. S. Roy; UAS-Rab5DN was kindly provided by M. Gonzalez-Gaitan. Other stocks used are: wgnKO; UASwgn-flag; egrDelta25; UAS-egr; UAS-puc and puc lacZ; btl-Gal4,UAS-srcGFP and UAS-sdk-V5 (generated in our laboratory).

Wild animals

This study did not involve wild animals

Reporting on sex

We obtained the data from large pools of embryos, which contained an equivalent proportion of both sexes, and we did not distinguish or selected male and female embryos

Field-collected samples

The study did not involve samples collected from the field

Ethics oversight

Drosophila studies are not subjected to ethical regulation

Note that full information on the approval of the study protocol must also be provided in the manuscript.
